# Biodegradation of Crude Oil and Aniline by Heavy Metal-Tolerant Strain *Rhodococcus* sp. DH-2

**DOI:** 10.3390/microorganisms12112293

**Published:** 2024-11-12

**Authors:** Zetian Luo, Jiajun Ma, Lei Huang, Dahui Li, Guohui Gao, Yihe Zhao, Agostinho Antunes, Meitong Li

**Affiliations:** 1 Tianjin Key Laboratory of Organic Solar Cells and Photochemical Conversion, College of Chemistry and Chemical Engineering, Tianjin University of Technology, Tianjin 300384, China; luozetian@stud.tjut.edu.cn (Z.L.); martintin@stud.tjut.edu.cn (J.M.); lidahui@stud.tjut.edu.cn (D.L.); gaoguohui@stud.tjut.edu.cn (G.G.); 2 CIIMAR/CIMAR, Interdisciplinary Centre of Marine and Environmental Research, University of Porto, Terminal de Cruzeiros do Porto de Leixões, Av. General Norton de Matos, s/n, 4450-208 Porto, Portugal; yihe.zhao@ciimar.up.pt (Y.Z.); aantunes@ciimar.up.pt (A.A.); 3 Department of Biology, Faculty of Sciences, University of Porto, Rua do Campo Alegre, s/n, 4169-007 Porto, Portugal

**Keywords:** aniline degradation, *Rhodococcus* sp., catechol pathway, crude oil, combined pollution

## Abstract

Aniline and crude oil are common environmental pollutants that present a significant risk to both the ecological and human health environments. The implementation of efficacious bioremediation strategies is imperative for the elimination of these contaminants. In this study, a bacterial strain designated DH-2 was isolated from soil contaminated with aniline. The strain was identified as belonging to the genus *Rhodococcus*. The optimal conditions for the growth and aniline degradation by strain DH-2 were determined to be pH 8.0 and 35 °C, respectively. Under these conditions, the degradation rate of aniline at a concentration of 1000 mg/L exceeded 90% within 36 h. Even in the presence of 4% NaCl, the degradation rate remained above 60%. HPLC–MS analysis revealed that the aniline degradation pathway of strain DH-2 follows the catechol pathway. Additionally, strain DH-2 is capable of utilizing crude oil as the sole carbon source, achieving a degradation rate of 91.0% for 2% crude oil concentration within 4 days. In soil modeling experiments, strain DH-2 was observed to degrade aniline and crude oil under triple stress conditions, including 1000 mg/L aniline, 2% crude oil, and 20 mg/L Fe(II) or Pb(II). Complete degradation of aniline and crude oil was achieved after 3 days and 12 days, respectively. The addition of Fe(II) or Pb(II) ions was found to enhance the degradation ability of DH-2. These results demonstrate that strain DH-2 is an extremely effective biodegradable strain, with potential applications in the remediation of environments contaminated with aniline and crude oil, even in the presence of heavy metals.

## 1. Introduction

Aniline is a crucial chemical intermediate, used in the industrial production of pesticides, dyes, plastics, pharmaceuticals, raw materials, and intermediates. It is a prevalent component in the manufacturing of printing and dyeing materials, pharmaceuticals, rubber, and other industrial wastewater [1]. The current global annual emission of aniline is estimated at approximately 30,000 tons, a figure that gives rise to considerable environmental concern. The high toxicity, large volume, and resistance to biological degradation of aniline wastewater present significant challenges in the field of environmental management. In the event of inadequate handling, there is a significant risk of substantial environmental contamination [2]. The tendency of aniline molecules to adhere to colloidal organic matter complicates their natural breakdown process, thereby increasing the risk of environmental pollution [3]. The primary environmental impact of aniline concentrate is its accumulation in soil and aquatic ecosystems, leading to subsequent pollution. Due to its volatile nature, aniline is capable of dispersing into the atmosphere as a vapor, thereby causing air pollution issues. Subsequently, aniline is transported into water bodies through atmospheric deposition or direct discharge [4,5]. This not only alters the physical and chemical properties of water but also poses potential hazards to aquatic biodiversity, disrupting the ecological balance of water systems. Moreover, aniline has the potential to permeate and accumulate in soils, leading to the establishment of persistent sources of pollution. Given its exceedingly slow rate of degradation in soil, contamination severely constrains the soil’s self-recovery capabilities, which has a detrimental impact on agricultural productivity and ecological health over long periods [6].

In addition to the challenges posed by soil contamination, the broader environmental implications associated with oil production further exacerbate these issues. As a significant source of energy and industrial raw material, the potential environmental impact of oil exploration, production, storage, refining, and consumption cannot be ignored. Currently, the amount of contaminated soil generated by oil operations in China’s principal oil fields exceeds 3 million tonnes per year, with the global total having risen markedly to approximately 60 million tonnes [7]. Petroleum-based pollutants are commonly released into the environment through the processes of crude oil exploration, extraction, transport, and refining. This results in the contamination of soil, groundwater, oceans, and even the atmosphere [8,9]. Once released into the environment, petroleum pollutants are highly resistant to degradation by natural processes. The cumulative effect of these pollutants over time will have a significant impact on the ecological balance, with serious consequences for biodiversity and indirect effects on human health through the food chain.

In view of the considerable number of industrial activities and wastewater discharges, the dispersion of pollutants in the natural environment has been observed to exhibit intricate composite patterns rather than isolated states [10,11]. It is particularly concerning that contamination by heavy metals (HM) is often intertwined with that of crude oil and aniline, which presents a significant environmental challenge. As indicated in the list of priority pollutants published by the United States Environmental Protection Agency (EPA) [12], approximately 40% of hazardous waste are a combination of HMs and organic pollutants. The presence of such mixtures significantly complicates the process of pollution control. The physical, chemical, and biological interactions between HMs and organic compounds are of particular significance in composite pollution systems [13]. For example, cation-π interactions represent a pivotal mechanism, which not only facilitates the formation of heavy metal–organic pollutant complexes but may also exert a synergistic toxic effect on microorganisms by altering the bioavailability and stability of the pollutants, which may compromise the efficacy of bioremediation-based technologies [14]. The toxicity of complex pollutants frequently exceeds that of single pollutants, representing a significant threat to the stability of environmental ecosystems and human health [15,16,17]. In light of this, there is an urgent need to develop efficient and sustainable strategies to address this environmental pollution challenge [18]. Microbial remediation is becoming increasingly popular among researchers due to its high efficiency, low cost, and lack of secondary contamination [19]. Microbial remediation represents a pivotal technique within the domain of environmental restoration. It entails the utilization of the metabolic activities of microorganisms for the purpose of degrading, transforming, or removing pollutants from the environment. This approach has been widely applied in the remediation of various types of pollution, including the degradation of organic contaminants [20], the restoration of heavy metal-contaminated sites [21], and the breakdown of pesticides and industrial chemicals [22]. Nevertheless, the extant literature suggests that the majority of microorganisms are efficacious in remediating single contaminants but less so in addressing complex composite contaminants [23,24]. This research aims to address these limitations by exploring novel avenues for the effective restoration of environments contaminated with complex mixtures of pollutants, specifically aniline, crude oil, and heavy metals.

In this study, the degradation capabilities of a *Rhodococcus* sp. strain isolated from a soil sample that has been long-term contaminated with aniline was investigated. The strain was noted to have the ability to grow when aniline served as the exclusive provider of both carbon and nitrogen. Moreover, the soil sample was found to contain a certain quantity of crude oil. Following experimental domestication, the obtained strain was also observed to be capable of growth with crude oil as the sole carbon source. This paper investigates the degradation performance of the strain on aniline, the laboratory simulation of the dual stress of aniline and crude oil in soil, and the degradation effect in the presence of heavy metal contamination and the dual stress of aniline and crude oil in soil. Additionally, the gene expression produced by the strain when utilizing aniline or crude oil as a single energy source is explored. This provides a fundamental theoretical foundation for the utilization of this bacterium in the bioremediation of aniline and crude oil dual-pollution environments. This study has the potential to serve as an effective tool for addressing complex pollution issues, particularly in areas in close proximity to industrial zones or oil extraction sites, where the presence of multiple pollutants is frequently observed.

## 2. Materials and Methods

### 2.1. Chemicals and Reagents

Aniline (97% purity) was obtained from Shanghai Senhua Technology Co., Ltd. (Shanghai, China), methylene chloride (98% purity) from Tianjin Standard Chemical Reagent Co., Ltd. (Tianjin, China), and glutaraldehyde (50% purity) from Tianjin Fuchen Chemical Reagent Factory (Tianjin, China). The crude oil used in the study was sourced from the Qizhong area of the Karamay Oilfield, located in Xinjiang, China. All other reagents were of analytical grade and were purchased from Tiangen Biochemical Co., Ltd. (Beijing, China).

The composition of the medium is displayed in Supplementary Section S1 (Supplementary Information).

### 2.2. Isolation and Identification of the Predominant Strain 

The aniline degradation strain was enriched and isolated in an area of China with a high concentration of aniline contamination, as determined by prior research [25,26]. One gram of aniline-contaminated soil sample was transferred into 30 mL of inorganic salt medium and incubated at 200 rpm/min and 30 °C for 72 h. The bacterial suspensions were subjected to four consecutive rounds of enrichment and the concentration of aniline was increased by 500 mg/L during each round of enrichment, with the resulting supernatants diluted to 10^−3^, 10^−5^, 10^−7^, and 10^−9^, respectively. A 100-microlitre aliquot of each dilution was plated onto solid Luria–Bertani (LB) medium and incubated in a biochemical incubator at 30 °C until colonies were visible. The colonies were then purified into single colonies through repeated streaking. Subsequently, single colonies were selected for inoculation into an inorganic salt medium (without nitrogen source) containing 50 mg/L aniline to verify the strain’s ability to grow using aniline as the sole carbon and nitrogen source. The strain exhibiting the fastest growth rate was selected for further investigation as the research strain. The strain was identified at the genus and species level through 16S rDNA analysis [27]. The extraction of microbial genomes and the identification of strains are presented in Supplementary Section S2.

### 2.3. Degradation Characteristics of Aniline-Degrading Bacteria

The incubation was carried out at 30 °C with a shaking speed of 200 rpm to ascertain the extent of aniline degradation. The absorbance at 600 nm (Abs) (Shanghai Spectrum Instruments Co., Ltd., Shanghai, China) was employed to monitor cell growth, while the rate of aniline degradation was determined using gas chromatography (GC) (BRUKER 456-GC, Billerica, MA, USA).

Aniline metabolic intermediates were subjected to analysis using High-Performance Liquid Chromatography–Mass Spectrometry (HPLC–MS) (Waters Acquity UPLC Class I/Xevo GQ—Tof, Waters, Shanghai, China). The intermediate metabolites were identified by comparing the relative molecular masses obtained at different retention times under identical experimental conditions. The detailed chromatographic conditions for the analysis of aniline are provided in Supplementary Section S3.

### 2.4. Crude Oil Degradation Experiment

The seed solution was inoculated into a crude oil degradation medium at a concentration of 2% in order to examine the degradation of crude oil. The crude oil, dissolved at a final concentration of 2% n-hexane was first added to a 100 mL conical flask, and then the triangular flask was shaken at 200 rpm and 35 °C for 2 h, with the objective of volatilizing the n-hexane. After removing the n-hexane, a film of crude oil was formed on the surface of the conical flask, and then 30 mL of inorganic salt medium was added for sterilization. After inoculation, the flasks were subjected to incubation at 35 °C with agitation at 200 rpm for 96 h to detect the degradation of crude oil. The degradation rate of the crude oil was determined by GC. The methodology employed was based on that described in the literature [28], where the residual oil was extracted twice with 30 mL of n-hexane containing 0.006% squalane. The organic phases of the two extracts were pooled, dehydrated using anhydrous Na_2_SO_4_ (approximately 1–2 g of anhydrous Na_2_SO_4_ was added to 10 mL of the liquid sample), and the solvents were subsequently evaporated through rotary evaporation. The residue was dissolved in an appropriate amount of n-hexane. The residual crude oil content was determined by GC. To ascertain the actual degradation rate of the crude oil following extraction, a control test was conducted utilizing the same crude oil concentration, with a sterile control medium devoid of bacterial suspension. The rate of degradation of the crude oil was calculated according to the following formula [29]:

Degradation rate of crude oil = (C1 − D2)/C1 × 100%

where C1 is the crude oil content in the control experiment after t days, %; and D2 is the crude oil content in the degradation experiment after 96 h, %.

### 2.5. DH-2 Whole Genome Sequencing 

The bacterial genome was extracted using a bacterial genomic DNA extraction kit in accordance with the instructions provided. After passing the gene quality check, the DNA samples were interrupted using an ultrasound, and the purified DNA was collected for library construction and on-board sequencing [30,31]. Sequencing was performed using the Illumina platform and genome scans were assembled according to the method of Luo [32]. The predicted Coding Sequences (CDSs) were functionally annotated using sequence alignment tools such as BLASTP, Diamond, and HMMER against databases including NR, Swiss-Prot, Pfam, GO, COG, and KEGG. This Whole Genome Shotgun project has been deposited at DDBJ/ENA/GenBank under the accession JBFSFC000000000. The version described in this paper is version JBFSFC010000000.

### 2.6. Fluorescence Quantitative Real-Time Polymerase Chain Reaction

Real-time fluorescence quantitative PCR was performed using SsoFast EvaGreen Supermix (BioRad, Hercules, CA, USA). First, 10 mL of liquid LB culture of strain DH-2 was taken and incubated in the presence of different carbon sources (ethanol, aniline, n-16 alkane) at 37 °C (200 rpm with shaking) for 72 h. The RNAprep pure cell/bacteria kit (TianGen, Beijing, China) was used to extract total RNA. The total RNA concentration of the isolated material was determined using a Qubit spectrophotometer (ThermoFisher, Waltham, MA, USA). Subsequently, cDNA product was generated using a Toyobo qPCR kit with 500 ng RNA as a template. The expression of four genes (*cadA1*, *cadA2*, *catA*, and *p450*) and 16S gene (reference gene) was detected by quantitative PCR apparatus with IQTMSYBR^®^GreenSupermix (BioRad, Hercules, CA, USA), and the fold change in gene expression was calculated by 2^−ΔΔCT^ [33]. The primer sequences used for each gene are provided in Table S2. 

### 2.7. Effect of Heavy Metal Ions on the Growth of Bacterial Strains

Stock solutions of Fe(II), Pb(II), Cd(II), Cr(VI and III), Zn(II), Ni(I), and Cu(II) were prepared using the following compounds: MnSO_4_·H_2_O, CdCl_2_·2.5H_2_O, CuCl_2_·2H_2_O, FeSO_4_·7H_2_O, ZnSO_4_·7H_2_O, NiCl_2_·6H_2_O, Pb(NO_3_)_2_, CrCl_3_·6H_2_O, and K_2_Cr_2_O. The final concentration of heavy metal ions in the mother solution was 10 mg/mL. Then, varying volumes of the metal ion stock solutions were added to the seed culture medium to achieve final concentrations ranging from 5 to 30 mg/L. Following a 60 h incubation period, the OD_600_ values were measured to assess the growth of the strain. Subsequently, the heavy metal ions that promoted strain growth were identified, and their effects on the strain’s growth were further examined using scanning electron microscopy (SEM) to observe any morphological changes.

### 2.8. Soil Simulation Experiment for the Treatment of Dual Pollution of Aniline and Crude Oil in the Presence of Heavy Metals

As indicated in Table 1, multiple treatment groups were designed, each with three biological replicates. All treatments were conducted under controlled conditions of 35–37 °C and a relative humidity of 60%, ensuring that the soil moisture content was maintained between 10% and 15%. The entire experimental period was set to 12 days [34].

### 2.9. Statistical Analysis

Significant differences were evaluated using Student’s *t*-test and analysis of variance (ANOVA), followed by the Tukey–Kramer post hoc test where significant effects were detected. A *p*-value less than 0.05 was considered statistically significant. All data are presented as mean ± standard deviation (SD). Experiments were conducted in triplicate or more to ensure reproducibility.

## 3. Results 

### 3.1. Aniline Degradation by Rhodococcus sp. DH-2

#### 3.1.1. Classification and Identification of Aniline Degrading Bacteria

**Figure 1:**
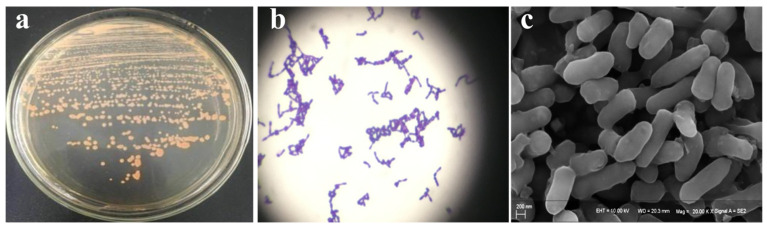
Morphology of DH-2 colonies (**a**), Gram staining (**b**), and SEM observation (**c**).

Through enrichment, culture, isolation, and purification, a high-efficiency aniline degradation bacteria, designated as DH-2, was isolated from an aniline-contaminated soil sample. The bacterial strain was observed to be capable of growth with aniline as the sole carbon and nitrogen source. Through colony morphology, Gram staining, and scanning electron microscope observation (Figure 1), it was determined that the bacteria were Gram-positive, short rod-shaped, without spores, with a typical eight-shaped arrangement. The colony surface was observed to be smooth, orange, opaque, and convex. The physiological and biochemical characteristics are shown in Table S1.

**Figure 2:**
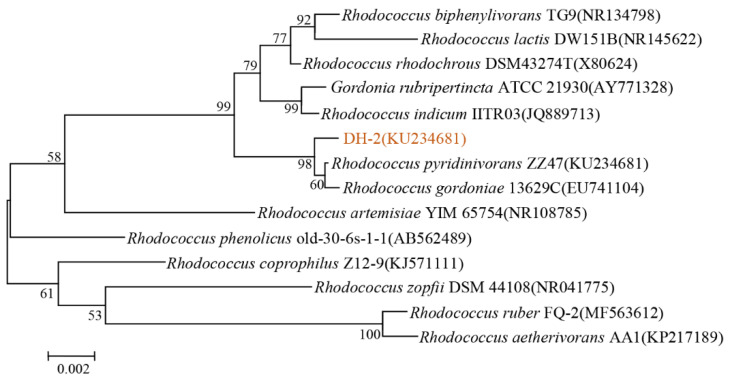
Phylogenetic tree between strains based on 16S rDNA sequence homology.

Strain DH-2 showed 99% homology with *Rhodococcus pyridinivorans* ZZ47 and *Rhodococcus gordoniae* 13629C. However, the physiological and biochemical characteristics (Table S1) demonstrated that strain DH-2 was consistent with *Rhodococcus gordoniae* and differed significantly from *Rhodococcus pyridinivorans*. Based on the physiological and biochemical characteristics of strain DH-2 and the results of the 16SrDNA sequence analysis, strain DH-2 was initially identified as *Rhodococcus gordoniae*. and named *Rhodococcus* sp. DH-2 (Figure 2).

#### 3.1.2. Influence of Environmental Conditions on the Degradation of Aniline by DH-2

**Figure 3:**
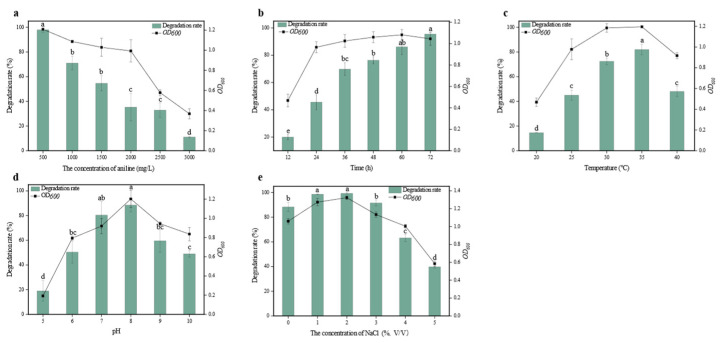
Effects of environmental conditions on the growth of DH-2 and its ability to degrade aniline. (**a**) Concentration of aniline, (**b**) Culture time, (**c**) Temperature, (**d**) pH, (**e**) Concentration of NaCl. The data presented in the graph are the mean ± SD values of replicates. The different letters above the bars indicate significant differences among the different groups (*p* < 0.05, *n* = 3). Statistical significance was determined using Tukey–Kramer test.

The results presented in Figure 3 demonstrate that strain DH-2 is capable of effectively degrading aniline at concentrations ranging from 500 to 1500 mg/L, with degradation rates exceeding 50% after 48 h of shaking culture. This suggests that the strain has significant potential for practical applications. Specifically, at a concentration of 500 mg/L, aniline was almost completely degraded, while the degradation rate for 1000 mg/L aniline was 71%. Accordingly, 1000 mg/L aniline was identified as the baseline concentration for subsequent optimization of environmental conditions. Subsequent experiments demonstrated that in an inorganic salt medium with 1000 mg/L aniline as the sole carbon and nitrogen source, the degradation rate of strain DH-2 reached 69.8% after 36 h of shaking culture at 30 °C. It was observed that as the culture time increased, the degradation rate declined. Accordingly, 36 h was selected as the optimal culture duration for subsequent optimization. It was determined that temperature has a considerable effect on the growth and aniline degradation capabilities of strain DH-2. At 35 °C, the degradation rate reached 82%, indicating that this temperature is optimal for the strain’s activity. Following the optimization of the environmental conditions, it was determined that 35 °C was the optimal temperature for culturing the organism. The findings indicated that strain DH-2 demonstrated the capacity to effectively degrade aniline across a broad pH range (6–10) within a 36 h period, with degradation rates exceeding 50%. This indicates that the strain is appropriate for the remediation of alkaline environmental contamination. At pH 5, both growth and degradation were significantly inhibited, while the highest degradation rate of 88.5% was observed at pH 8. Finally, the experiments demonstrated that salinity is a critical environmental factor affecting microbial growth. The presence of elevated salt concentrations can result in water loss and cell contraction, which in turn impedes growth and metabolic processes. The growth and degradation of strain DH-2 were observed to be higher than those of the control group in the presence of 1–3% NaCl. Furthermore, the degradation rate at 4% NaCl exceeded 60%. Although 5% NaCl inhibited bacterial growth, the degradation rate remained at 40.3%, indicating that strain DH-2 exhibits moderate salt tolerance. The highest degradation rate of 99.3% was observed at a NaCl concentration of 2%, indicating that low to moderate salt concentrations have a beneficial effect on the strain’s performance.

#### 3.1.3. Analysis of the Degradation Pathway of Aniline by Strain DH-2

**Figure 4:**
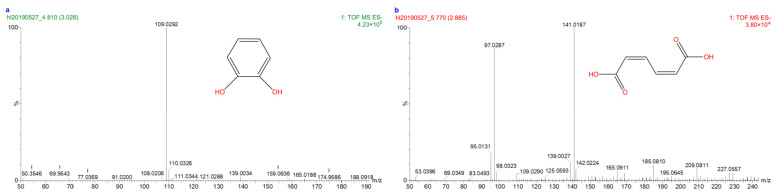
The intermediate products of aniline degradation by strain DH-2 were analyzed by LC-MS. (**a**) catechol; (**b**) *cis*,*cis*-muconic acid.

The degradation of aniline by microorganisms is primarily achieved through the catechol pathway. Prior research [35] has demonstrated that aniline is first transformed into catechol by aniline dioxygenase, which is then further degraded by catechol 1,2-dioxygenase and catechol 2,3-dioxygenas. The degradation pathway of aniline by strain DH-2 was analyzed using HPLC–MS. The extracted intermediates were subjected to comparative analysis, which led to the identification of two metabolic intermediates, catechol and cis,cis-mucoconic acid. These are illustrated in Figure 4. The absence of 4-hydroxy-2-oxo-pentanol indicates that the enzyme responsible for the conversion of aniline to catechol in strain DH-2 is catechol 1,2-dioxygenase rather than catechol 2,3-dioxygenase.

#### 3.1.4. Genomic Analysis of *Rhodococcus* sp. DH-2

**Figure 5:**
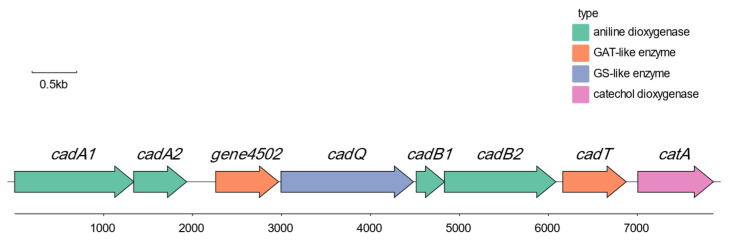
Gene cluster of aniline degradation genes in strain DH-2’s genome.

**Figure 6:**
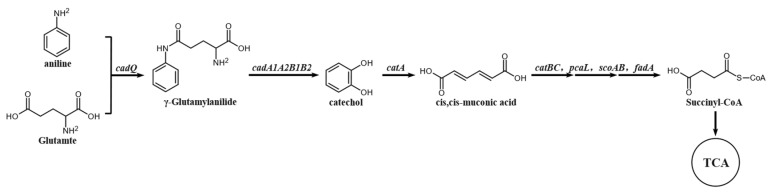
Pathway of aniline degradation by DH-2.

Whole-genome sequencing holds the potential to efficiently uncover the key genes associated with aniline degradation by strain DH-2. Among the annotated 4576 genes of *Rhodococcus* sp. DH-2, 150 genes was identified as being involved in xenobiotic biodegradation. A cluster of genes associated with aniline degradation was identified within the genome, with a length of 7855 bp (Figure 5). This cluster exhibited high similarity to the aniline degradation gene cluster of *Rhodococcus* sp. AN-22, as previously reported by Matsumura et al. [36]. The genome contains seven degradation genes, *cadA1*, *cadA*, *cadB1*, *cadB2*, *cadQ*, *cadT*, *catA*, which encode the aniline dioxygenase big subunit, aniline dioxygenase small subunit, [2Fe-2S] ferredoxin, aniline dioxygenase reductase, glutamine synthetase(GS)-like enzyme, glutamine amidotransferase(GAT)-like enzyme, and catechol 1,2-dioxygenase. This gene cluster can degrade aniline into a cis,cis-muconic acid, which is consistent with our results from the LC–MS. Additionally, we identified several genes in the genome of *Rhodococcus* sp. DH-2, including *catB*, *catC*, *pcaL*, *scoA*, *scoB*, and *fadA*. These genes encode the following enzymes: muconate cycloisomerase, muconolactone D-isomerase, 3-oxoadipate enol-lactonase, 3-oxoacid CoA-transferase subunit A, 3-oxoacid CoA-transferase subunit B, and acetyl-CoA acyltransferase, respectively. These enzymes further degrade the metabolic product cis,cis-muconic acid into Succinyl-CoA, which can then enter the TCA cycle, leading to the complete mineralization of aniline. The entire degradation pathway of aniline is illustrated in Figure 6.

### 3.2. Degradation Efficacy of DH-2 in Crude Oil Contaminated Environment

#### 3.2.1. Degradation of Crude Oil by Strain DH-2

**Figure 7:**
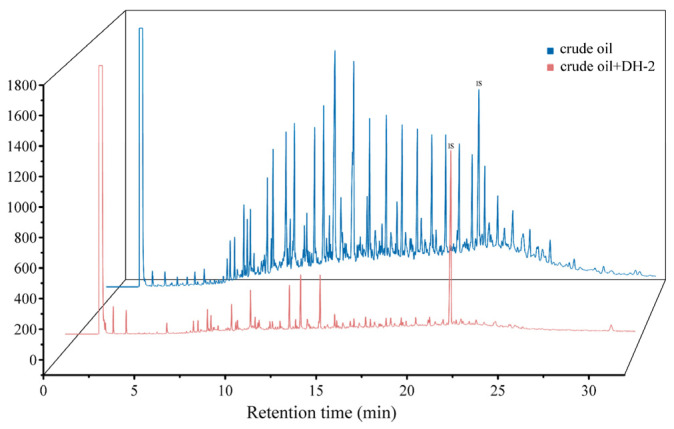
Results of GC analysis of crude oil degradation by DH-2.

The crude oil subjected to the DH-2 extraction process was subsequently extracted with hexane, and the resulting degradation was analyzed by gas chromatography (GC) using squalane as the internal standard (IS). As illustrated in Figure 7, the degradation rate of strain DH-2 for a 2% mass fraction of crude oil at 35 °C for 4 d was 91.0%, calculated from the GC peak area. The gas chromatograms showed that strain DH-2 exhibited a notable degradation effect on most of the components present in the crude oil. 

#### 3.2.2. Fluorescence Quantitative Real-Time Polymerase Chain Reaction (qPCR) Analysis

**Figure 8:**
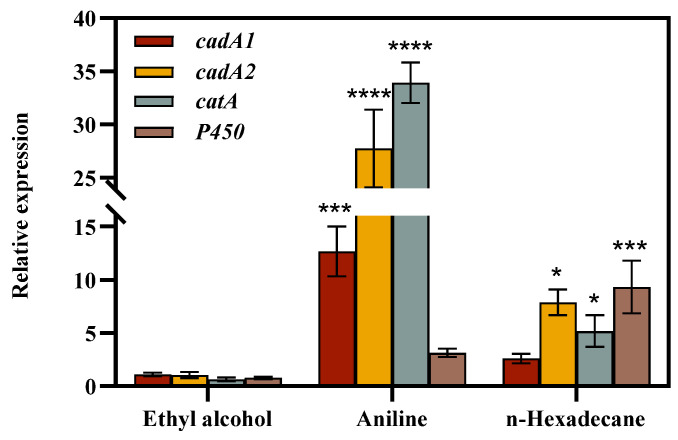
Expression levels of related genes. The data presented in the graph are the mean ± SD values of replicates. Significant differences were assessed by Student’s *t* test analysis (* *p* < 0.05, *** *p* < 0.001, **** *p* < 0.0001).

The following our genes associated with degradation were selected for transcript level determination: *cadA1*, *cadA2*, *catA*, and *P450*. The *cadA1* and *cadA2* genes encode large- and small-subunit oxygenases, respectively, which are the primary enzymes involved in the degradation of aniline. The *catA* gene encodes catechol 1,2-dioxygenase, which is responsible for the degradation of catechol within cells. The *P450* gene encodes cytochrome monooxygenase, which is involved in a range of metabolic processes and can be utilized for the cellular degradation of alkanes and hydrocarbon pollutants. Some studies have shown that *P450* may also play a role in the degradation of aromatic amines [37]. It was postulated that the discrepancies in gene expression may be attributable to the disparate types of carbon sources employed as substrates. The expression of the *cadA1*, *cadA2*, *catA*, and *P450* genes was markedly elevated by strain DH-2 at 72 h under the conditions of 1000 mg/L aniline and 2% crude oil, respectively (Figure 8). Significantly, at a concentration of 1000 mg/L aniline, the expression of the *catA* gene was markedly elevated, indicating that strain DH-2 degraded aniline via the catechol 1,2 dioxygenase pathway, which is consistent with the previous conclusion. The presence of 1000 mg/L aniline and 2% crude oil was observed to upregulate the expression of *P450* genes, with the effect of crude oil being more pronounced. This suggests that the *P450* has an impact on the degradation of crude oil and aniline, with a more pronounced effect on the latter.

### 3.3. Remediation of Aniline–Crude Oil–Heavy Metal Triple Contaminated Soil by DH-2

#### 3.3.1. The Impact of Heavy Metals on the Growth of DH-2

As demonstrated in Table 2, the minimum inhibitory concentration (MIC) of strain DH-2 was found to be greater than 30 mg/L for Fe(II) and Pb(II), and 20 mg/L for Mn(II) and Cd(II). The MIC for Cr(VI, III), Zn(II), and Ni(I) was 15 mg/L. However, no MIC values were obtained for Cu(II).

The addition of Pb(II) to the seed medium at low concentrations (0–20 mg/L) had a negligible impact on DH-2 cell growth. However, an increase in Pb(II) concentration above 25 mg/L was observed to result in a decline in cell growth. The findings indicated that Fe(II) was the least toxic heavy metal to the DH-2 strain, and that the strain exhibited the greatest tolerance to it. The growth of the strain pairs in the presence of heavy metal ions was ranked in the following order: The order of toxicity was Fe(II) > Pb(II) > other heavy metals.

As shown in Figure S2, the growth of the strains was examined at various time points following the addition of varying heavy metal ions to the seed medium at a concentration of 20 mg/L. The addition of heavy metal ions other than Fe(II) and Pb(II) resulted in a reduction in the growth of the bacterium. Of these, Cu(II) was identified as the most toxic heavy metal to the DH-2 strain, exhibiting the most significant inhibitory effect on bacterial growth. The bacterium demonstrated robust tolerance to Fe(II) and Pb(II), exhibiting enhanced growth compared to the control after 48 h and 24 h in the presence of Pb(II) and Fe(II), respectively. This indicates that these two heavy metals exert a beneficial effect on the strain. The SEM images of DH-2 (Figure S3) demonstrate that the bacterial cell surface exhibits rough and wrinkled characteristics when exposed to Pb(II) and Fe(II) ions. This observation suggests that the tolerance mechanism of bacteria to heavy metals may involve the chelation of metals, surface adsorption, the precipitation of organic functional groups [38], as well as changes in the cell surface morphology due to the deposition of heavy metal ions [39]. Despite the modifications to the cell surface morphology caused by the presence of heavy metal ions, no significant damage or structural disruption was discerned in the SEM images. This indicates that DH-2 bacteria are capable of effectively coping with the challenge posed by heavy metal ions through the aforementioned mechanisms, thereby maintaining the integrity of their cellular structure. Therefore, it can be inferred that Pb(II) and Fe(II) do not significantly inhibit the growth of DH-2 bacteria, indicating a high level of resistance to heavy metals.

#### 3.3.2. Soil Modelling Experiments for the Treatment of Dual Aniline and Crude Oil Contamination in the Presence of Heavy Metals

**Figure 9:**
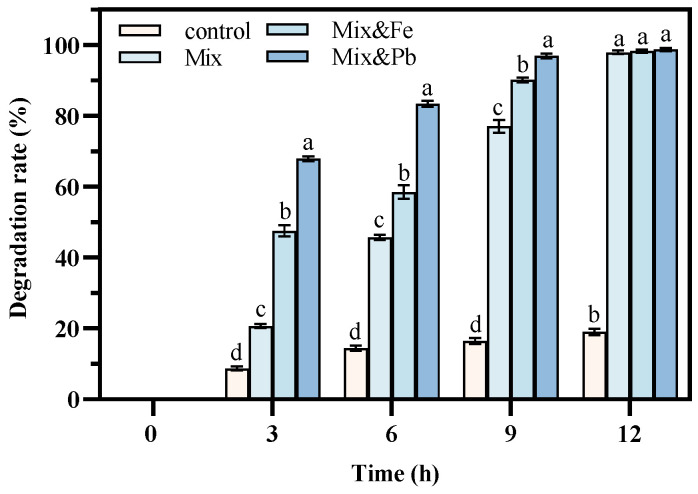
Degradation rate (%) in co-contaminated soil (all soils had a concentration of aniline 1000 mg kg^−1^ and crude oil 200 g kg^−1^ dry soil on day zero) (Treatment 1, sterilized soil + aniline + crude oil; Treatment 2, sterilized soil + aniline + crude oil + DH-2; Treatment 3, sterilized soil + aniline + crude oil + Fe(II) + DH-2; Treatment 4, sterilized soil + aniline + crude oil + Pb(II) + DH-2). The data presented in the graph are the mean ± SD values of replicates. The different letters above the bars indicate significant differences among the different groups (*p* < 0.05, *n* = 3). Statistical significance was determined using Tukey–Kramer test.

Figure 9 illustrates that strain DH-2 exhibited effective degradation under optimal culture conditions, comprising 1000 mg/L aniline, 2% crude oil, and 20 mg/L Fe(II) and Pb(II), in the context of a double pollution. Given that aniline was completely degraded within 3 d, the degradation rate of crude oil was employed to characterize the degradation ability of strain DH-2. The complete degradation of the crude oil was observed after 12 d, which was facilitated by the presence of Fe(II) and Pb(II) ions. The degradation efficiency of aniline with the addition of 20 mg/L Fe(II) was found to be inferior to that observed with the addition of 20 mg/L Pb(II). Furthermore, the oxidation of Fe(II) in the air was readily observed, and the specific mechanism underlying this phenomenon requires further investigation. In these conditions, strain DH-2 was still able to degrade aniline and crude oil effectively, indicating that it is a superior candidate for the bioremediation of environments contaminated with aniline, crude oil, and heavy metals. It also has the potential for application.

## 4. Discussion 

### 4.1. A High-Effective Crude Oil and Aniline Degrading Strain Was Isolated and Identified

The complexity of pollution serves to exacerbate global environmental problems, thereby posing a significant threat to the continued survival of humans and all living organisms. Aniline is a chemical carcinogen that can be degraded by a wide range of microorganisms. However, it is widely present in the environment [35] and is often found in contaminated soils, sediments, or water in the vicinity of oil, coal, and power plants. Crude oil represents the world’s major energy source, but spills, whether at sea or on land, have the potential to cause long-term ecological damage. Crude oil comprises a complex mixture of organic compounds, including polycyclic aromatic hydrocarbons (PAHs), which are toxic to microorganisms and challenging to degrade. The utilization of microbial remediation has emerged as the principal methodology for the remediation of environmental contamination, offering a sustainable and cost-effective technological solution.

As a widely distributed and effective microbial remediation agent, *Rhodococcus* has been the subject of increasing attention with regard to its potential applications in bioremediation. *Rhodococcus* has been demonstrated to possess remarkable degradation capabilities with regard to both aniline and crude oil pollutants. Martina Cappelletti [40] explored the influence of different genetic structures of *Rhodococcus* monooxygenases on the regulatory mechanisms governing alkane degradation. Jia [41] isolated and selected a strain of *Rhodococcus* T1 and conducted a study on its biodegradation of n-hexadecane and pyrene, as well as its metabolic mechanism. Shintani [42] investigated the synergistic degradation effect of four *Rhodococcus* strains on A-fuel oil. Pham [43] isolated a cold-tolerant *Rhodococcus* sp. Y2-2 and subsequently studied its crude oil degradation characteristics in liquid and soil media. Our laboratory [28] had previously screened a *Rhodococcus* sp. HL-6 capable of utilizing No. 0 diesel as its sole carbon source, achieving a degradation rate of 78.5% within 3 days following optimization. Ma [44] made immobilized microspheres of *Rhodococcus* sp. PB-1 which were capable of completely removing 1500 mg/L aniline in 62 h. The simultaneous presence of aniline and crude oil organic pollutants presents a significant challenge to pollution control. Despite the abundance of research on individual microbial remediation techniques for aniline and crude oil pollution, there is a paucity of reports on the co-remediation of these two pollutants. However, the *Rhodococcus* sp. strain DH-2, which was the subject of this study, demonstrated the capacity to completely degrade 1000 mg/L of aniline within 3 days and showed effective degradation ability for 2% crude oil.

### 4.2. Degradation Genes and Enzymes of Crude Oil and Aniline Were Explored and Verified

The biodegradation of pollutants was mainly catalyzed and regulated by enzymes. Prior studies [35] have shown that the biodegradation pathway of aniline can be subdivided into two stages—the transformation of aniline to catechol, followed by the degradation of catechol. The conversion of aniline to catechol is responsible for the aniline dioxygenase (AD) gene cluster. All AD gene clusters are composed of three components. The aforementioned proteins include GS-like, GAT-like, and aniline dioxygenase (which contains both oxygenase (large and small) and ferredoxin-reductase). While the composition of the AD gene cluster remains consistent across all strains to date, there are notable differences in their substrate specificity. The discrepancy in substrate specificity was demonstrated by Ji [45] to be contingent upon GS-like proteins and oxygenases, thereby offering insights into the enzymatic engineering of AD gene clusters for modification. The ability of an aniline-degrading bacterium to fully mineralize aniline is contingent upon whether it has an intact cluster of catechol metabolism genes. The degradation of catechol can occur in three different forms, which have been designated as ortho-metabolic, meta-metabolic, and facultative [46,47]. The meta-metabolic pathway is catalyzed by catechol 1,2-dioxygenase, whereby catechol is degraded to cis, cis-mucoconic acid, which is subsequently further degraded in the TCA cycle. Catechol 2,3-dioxygenase functions as the catalyst for the catechol meta-metabolic pathway, whereby catechol is degraded to 4-hydroxy-2-oxo-pentanol. Subsequently, the compound undergoes an additional oxidative cleavage, resulting in the formation of pyruvate and acetaldehyde. These two intermediates then enter the TCA cycle directly.

The genome of the aniline-degrading bacterium *Rhodococcus* sp. DH-2 identified in this study contains a complete cluster of AD genes with catechol 1,2-dioxygenase, demonstrating its potential for the complete mineralization of aniline. The absence of catechol 2,3-dioxygenase in the genome of *Rhodococcus* sp. DH-2 indicates that the pathway of catechol degradation in this strain is ortho-metabolic. Two intermediates of aniline degradation, namely catechol and cis,cis-mucoconic acid, were identified by HPLC–MS analysis. However, 4-hydroxy-2-oxo-pentanol was not detected, which corroborates our initial hypothesis. In addition, the expression levels of the relevant genes were investigated through RT-qPCR, which revealed a notable upregulation of *cadA1*, *cadA2*, and *catA* in the presence of aniline. This demonstrates that strain DH-2 possesses a keen sensing mechanism that can rapidly respond to the signals of aniline in the environment and activate the corresponding degradation pathways thus efficiently removing this deleterious pollutant.

### 4.3. Analysis of the Degradation of Triple Contamination of Aniline, Crude Oil and Heavy Metals in Soil

The rapid expansion of industrialization and economic activities has precipitated an unprecedented threat to soil quality [48]. Globally, it is estimated that some 10 million hectares of land worldwide are contaminated to varying degrees [49]. These contaminated sites are often not the result of a single pollutant, but rather a combination of multiple inorganic and organic pollutants, including HMs, PAHs, pesticides, and antibiotics [11]. The presence of multiple metal ions at contaminated sites often results in complex effects on local microbial metabolic processes. Therefore, when implementing bioremediation strategies, it is particularly important to screen for strains that possess a high degree of heavy metal tolerance and are capable of coping with a wide range of metal species. The DH-2 strain is an optimal candidate, exhibiting a broad spectrum of tolerance properties, particularly towards Fe(II) and Pb(II). It is noteworthy that, in contrast to the commonly observed inhibitory effects of certain metals on microbial growth, some metals at low to moderate concentrations can stimulate the growth potential of the strain and enhance its degradation efficiency of the pollutants. For instance, *Alcaligenes* sp. j08 has been shown to effectively degrade aromatic compounds under the stress of 0.5 mm Cd(II) [50]. The addition of *Bacillus thuringiensis* FQ1 to soils treated with 300–500 mg/kg of Cd results in an increase of more than 75% in the biodegradation efficiency of aromatic compounds within a three-month period [50]. Similarly, the biodegradation of aromatic compounds is augmented in soils co-contaminated with aromatic compounds and 140 mg/kg of Zn(II) [51]. Strain DH-2 in this study also showed such characteristics; its growth was significantly promoted at lower concentrations of Fe(II) or Pb(II) and, in the simulated soil remediation experiments, Fe(II) and Pb(II) not only enhanced the activity of strain DH-2 but also significantly accelerated the degradation rate of aniline and crude oil in the soil. These findings underscore the exceptional bioremediation capabilities of strain DH-2 when confronted with composite pollution from aniline, crude oil, and heavy metals, suggesting the substantial potential for practical application in real-world soil bioremediation projects.

## 5. Conclusions

The objective of this study was to investigate the growth conditions of the aniline-degrading strain *Rhodococcus* sp. DH-2. The results demonstrate that this strain displays remarkable environmental adaptability, enabling efficient aniline degradation under diverse conditions. Furthermore, *Rhodococcus* sp. DH-2 exhibits robust growth and degradation capabilities in environments contaminated with aniline, crude oil, and heavy metals. It may therefore be considered an ideal candidate for the microbial remediation of aniline pollution and complex contaminated environments. These findings provide crucial insights for the future development of effective bioremediation technologies and have significant implications for the improvement in contaminated environments.

## Figures and Tables

**Table 1:** Soil preparation.

Group	1	2	3	4
Aniline (mg/kg dry weight soil)	1000	1000	1000	1000
Crude oil (g/kg dry weight soil)	200	200	200	200
Fe(II) (mg/kg dry weight soil)	—	—	20	—
Pb(II) (mg/kg dry weight soil)	—	—	—	20
DH-2 (D)/abiotic (A)	A	D	D	D

**Table 2:** Analysis of heavy metal ion tolerance of DH-2 strain with ethanol as the only carbon source.

Metal Ions	Metal Ion Concentration (mg/L)							MIC ^a^ (mg/L)
	0	5	10	15	20	25	30	
Zn(II)	++++	++	++	-	-	-	-	15
Cd(II)	++++	++	++	++	+	-	-	20
Fe(II)	++++	++++	++++	+++++	+++++	++++	+++	>30
Pb(II)	++++	++++	++++	++++	++++	+++	++	>30
Cu(II)	++++	-	-	-	-	-	-	—
Cr(III)	++++	++	++	+	-	-	-	15
Cr(VI)	++++	++	++	+	-	-	-	15
Ni(I)	++++	++	++	+	-	-	-	15
Mn(II)	++++	++	++	++	+	-	-	20

^a^: OD_600_ less than 0.5; +: OD_600_ more than 0.5 and less than 0.7; ++: OD_600_ more than 0.7 and less than 0.9; +++: OD_600_ more than 0.9 and less than 1.1; ++++: OD_600_ more than 1.1 and less than 1.3; +++++: OD_600_ more than 1.3.

## Data Availability

The original contributions presented in the study are included in the article/Supplementary Materials, further inquiries can be directed to the corresponding authors.

## Supplementary Materials

The following supporting information can be downloaded at: https://www.mdpi.com/article/10.3390/microorganisms12112293/s1, Figures S1: Growth curve of DH-2 at 25 °C; Figures S2: Effects of different heavy metal ions on the growth of DH-2 strain; Figures S3: SEM of DH-2, (a) the control, (b) after Pb(II) adsorption, (c) after Fe(II) adsorption; Tables S1: Comparison of physiological and biochemical properties between DH-2 and *R. pyridinivorans* PDB9 and *R. gordoniae* W4937; Tables S2: Primers used in this study.
