# Supplementary material for: Biodegradation of Crude Oil and Aniline by Heavy Metal-Tolerant Strain Rhodococcus sp. DH-2
Source: Microorganisms. 2024 Nov 12;12(11):2293. doi: 10.3390/microorganisms12112293 (PMC11596033; doi:10.3390/microorganisms12112293)
Supplement: Supplementary file 1 [file microorganisms-12-02293-s001.zip › microorganisms-3285284-supplementary.pdf]

# [Supplementary information]

# Biodegradation of Crude Oil and Aniline by Heavy Metal-Tolerant Strain *Rhodococcus* sp. DH-2

**Zetian Luo¶, Jiajun Ma¶, Da-hui Li, Guohui Gao, Lei Huang*, Meitong Li***

**Affiliations:**

College of Chemistry and Chemical Engineering, Tianjin Key Laboratory of Organic Solar Cells and Photochemical Conversion, Tianjin University of Technology, China.

***Corresponding Author**

E-mail address: huanglei@tjut.edu.cn (L. Huang) https://orcid.org/0000-0002-3490-3393

E-mail address: tjutlmt@email.tjut.edu.cn (M.T. Li)

¶These authors contributed equally to this work.

**Supplementary Sections**

**Section S1 |** The composition of different mediums.

The LB medium comprised the following components ($g \cdot L^{-1}$): peptone 10, yeast powder 5, and sodium chloride 5. The pH was adjusted to a range of 7.0–7.2.

The minimal salt medium was prepared according to the following specifications ($g \cdot L^{-1}$):

The composition of the medium was as follows: $Na_2HPO_4$ 1.5, $KH_2PO_4$ 3.48, $(NH_4)_2SO_4$ 4, $MgSO_4$ 0.7, and yeast powder 0.01; pH 7.2.

The seed medium was supplemented with 2% ethanol, while the hydrocarbon degradation medium was supplemented with 2% (v/v) crude oil. The composition of the crude oil from the seventh zone of the Karamay oilfield was as follows: the relative content of saturated alkane was 65.75%, the relative content of aromatic hydrocarbon was 7.67%, the relative content of non-hydrocarbon was 10.85%, and the relative content of asphaltene was 15.72%.

The aniline degradation minimal salt medium was prepared as follows ($g \cdot L^{-1}$): $Na_2HPO_4$ 1.5, $KH_2PO_4$ 3.48, $MgSO_4$ 0.7, yeast powder 0.01, and 1000 mg/L aniline; with a pH of 7.2. All media was sterilized at 121 °C for 30 min.

**Section S2 |** The extraction of microbial genomes and identification of strains.

The bacterial genome extraction kit (BGI-LH-305-02, BGI Co., Ltd., Beijing, China) was used to extract total bacterial DNA. The 16S rRNA gene was amplified by PCR with bacterial universal primers (27F: 5'-AGAGTTTGATCCTGGCTCAG-3'; 1492R: 5'-CTACGGCTACCTTGTTACGA-3'). The PCR amplification conditions were as follows: 95 °C for 5 min, 30 cycles of 94 °C for 45 s, 55 °C for 30 s, 72 °C for 90 s, and with a final extension at 72 °C for 10 min. Primer synthesis and PCR product sequencing were completed by BGI Co., Ltd., Beijing, China. Sequencing results were analyzed by Nucleotide BLAST (https://blast.ncbi.nlm.nih.gov/), and the top twenty-three strains with reliability were selected and used in the MEGA 7.0 software (Mega

Limited, Auckland, New Zealand) to construct a phylogenetic tree using the Neighbor-Joining (NJ) method.

**Section S3 |** Aniline analyses by GC and HPLC-MS.

Aniline degradation rate was determined by GC; the parameters were set as follows: The inlet temperature was set to 200°C, the detector temperature was 280°C, and temperature program started at 40°C for 2 minutes, then increased to 105°C at a rate of 20°C/min, held for 1 minute, and subsequently rose to 260°C at a constant rate of 60°C/min, with a final hold for 2 minutes, with a carrier gas flow of 10 mL/min of $N_2$, 30 mL/min of $H_2$, and 300 mL/min of air. The split ratio was 100:1, and the injection volume was 5 μL.

Crude oil degradation rate was determined by GC; the parameters were set as follows: An HP-5 capillary column (30 m×0.32 mm ID length, 0.25 μm film thickness) was employed for the analysis of 10 μL of the organic phase. During the analysis, the temperature of the gas chromatographic column was maintained at 80 °C for one minute, after which it was increased to 290 °C at a rate of 10 °C per minute and held for ten minutes.

Aniline metabolic intermediates were analyzed using HPLC–MS. For analysis, the column used was a Shimazu ODS-3 column (5 μm, 4.6 × 250.0 mm; MetaChem Technologies) equipped with a guard column containing the same stationary phase and connected to a Waters 515 solvent delivery system. The degradation products were eluted and analyzed by mass spectrometry at a flow rate of 1 mL/min using a programmed methanol/water gradient as the solvent system. The mobile phase was maintained in a linear gradient for 45 min in a solution comprising 50% (vol/vol) to 95% (vol/vol) methanol and water, for a further 10 minutes in a solution comprising 95% (vol/vol) methanol and water, and for a final 5 minutes in a solution comprising 95% (vol/vol) to 50% (vol/vol) methanol and water.

**List of Figures and Tables**

**Fig. S1.** Growth curve of DH-2 at 25 °C.

**Fig. S2.** Effects of different heavy metal ions on the growth of DH-2 strain.

**Fig. S3.** SEM of DH-2, (a) the control, (b) after Pb (II) adsorption, and (c) after Fe (II) adsorption.

**Table S1.** Comparison of physiological and biochemical properties between DH-2 and *R. pyridinivorans* PDB9 and *R. gordoniae* W 4937.

**Table S2.** Primers used in this study.

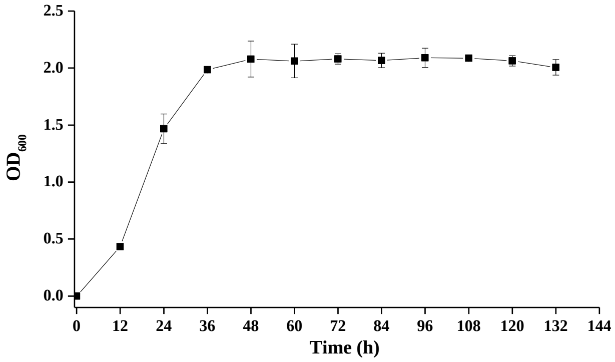


Figure S1. Growth curve of DH-2 at 25 ℃.

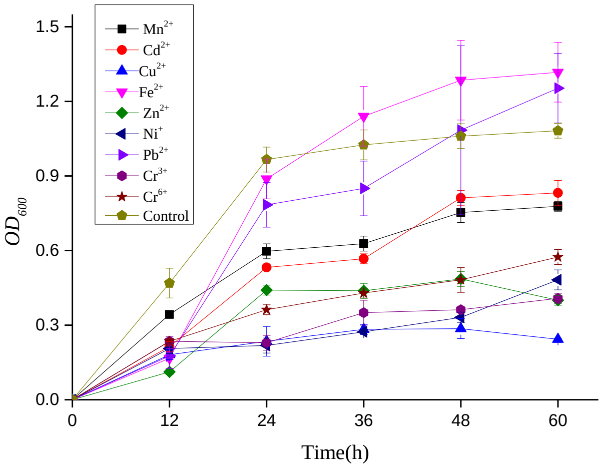


Figure S2. Effects of different heavy metal ions on the growth of DH-2 strain.

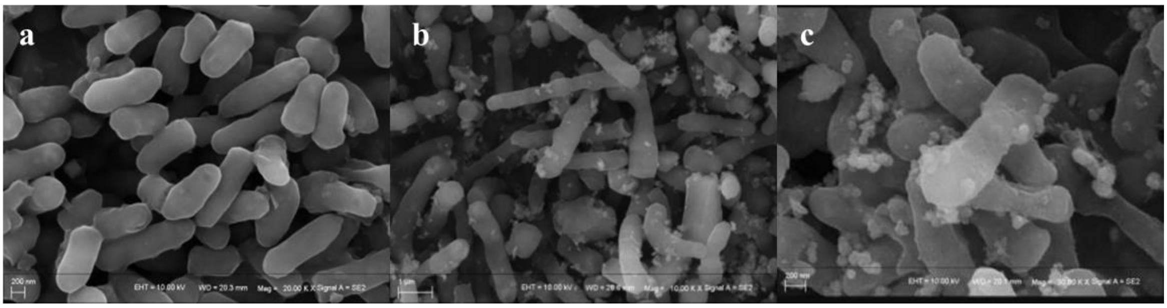

Figure S3. SEM of DH-2, (a) the control, (b) after Pb (II) adsorption, and (c) after Fe (II) adsorption.

Table S1. Comparison of physiological and biochemical properties between DH-2 and *R. pyridinivorans* PDB9 and *R. gordoniae* W 4937.

| Items | DH-2 | *R. gordoniae* W 4937 | *R. pyridinivorans* PDB9 |
|---|---|---|---|
| exposure enzyme | + | + | + |
| oxidase | − | − | − |
| Glucose fermentation for acid production | − | − | + |
| nitrate reduction | + | + | + |
| Citrate reduction | + | + | − |
| 6% NaCl growth | + | + | − |
| $H_2S$ | + | + | − |
| methyl red | − | − | ND |
| V-P | − | − | ND |
| amylum C6H10O5 | + | + | + |
| urease (enzyme) | + | + | − |
| Heptaphyllum hydrolysis | + | + | − |
| Pyridine degradation | + | + | ND |
| Indole production | − | − | − |

Note : “+” means positive, “-“ denotes negative, and “ND” means not detected.

Table S2. Primers used in this study.

| Primer | Primer sequence(5’-3’) |
|---|---|
| *cadA1*-F | AGCGTAACCTTCTTCAGATTG |
| *cadA1*-R | CGGAGTCGTCAACATCGTTC |
| *cadA2*-F | TGGTTGGTCATCACCATCATC |
| *cadA2*-R | CCCAGAATCAGGACGAACAG |
| *catA*-F | TGGTACTCCTACGCCTACTG |
| *catA*-R | GCCACATATCCTGCCAGAG |
| *P450*-F | GTGGTCACGGACAACGGAAC |
| *P450*-R | CGAGTGCTTGGCGGAGATC |
| 16S-F | CTCTTTCAGCAGGGACGAAG |
| 16S-R | AACCGCCTACGAACTCTTTAC |
